# The American Congress on Tuberculosis

**Published:** 1903-08

**Authors:** 


### TEXAS MEDICAL JOURNAL.

AUSTIN, TEXAS.

A MONTHLY JOURNAL OF MEDICINE AND SURGERY.

EDITED AND PUBLISHED BY

F. E. DANIEL, M. D.

ASSOCIATE EDITORS:

WITTEN BOOTH RUSS, M. D., San Antonio, Texas.

P. M. PAYNE, M. D., Brownwood, Texas.

Published Monthly at Austin, Texas. Subscription price $1.00 a year in advance.

Eastern Representative: John Guy Monihan, St. Paul Building, 220 Broadway, New York City.

Official organ of the the West Texas Medical Association, the Houston District Medical Association, the Austin District Medical Society, the Brazos Valley Medical Association, the Galveston County Medical Society, and several others.

### THE AMERICAN CONGRESS ON TUBERCULOSIS.

**THE GREAT WHITE PLAGUE.**

Few persons other than sanitarians and statisticians have any adequate idea of the ravages of consumption. According to the United States census reports there died from consumption in the United States, in 1900, 111,000 persons, so far as reported. The registration districts in the States where records are kept and reports made embrace only about one-third of the population; that is, the rural population, as a rule, is not represented in these reports. A calculation based on this fact and on the given ratio would give us a total for 1900 of 145,000 deaths; and for the census year 1890, 154,000, or an average yearly of about 150,000. Thus in the decade between the census years there were in the United States one and a half million deaths from consumption.*

*Says Professor W. S. Carter (Trans. Texas State Medical Association 1902, p. 366-7): "It might be contended that this estimate is too high, as the registration area includes mostly the urban population, while the non registration area represents largely the rural districts. Against this argument it may be stated that the total number of deaths recorded, from all causes, for the non registration area was 526,425, although the population was nearly two-thirds that of the entire country (47,000.000), while the total number of deaths from

In the War of Secession there was a total loss of three-fourths of a million soldiers all told, both armies, from all causes. The war lasted, say, five years. It will be seen that at the present time as many persons are dying yearly from consumption as were lost yearly by both sides, from all causes, during that terrible conflict.

If the bubonic plague were raging in America, and four hundred people were dying daily, every day, all the time, year in and year out; or there was a war in progress on our soil with such a death loss, what would be the state of public sentiment? There would be a panic and a stampede. Every resource of science and skill, every influence and power and agency on the part of government—State, national and local—would be invoked and put into exercise to stop the fearful havoc. And yet, that is just what consumption is doing. It is killing four hundred and eleven people every day, every year—all the time, and no one seems to know it or to heed it except the sanitarian and the statistician, and they are powerless to prevent it.

Consumption is a disease easily preventable. It is in no sense contagious, nor is it hereditary, as it was once thought to be. It is, however, highly infectious, and therefore communicable from the sick to the well, the infection being the poisonous matter coughed up by the patient. To prevent the spread of the disease and the reinfection (auto-infection) of a person getting better or well, it is necessary to destroy this poison. It can be readily seen that if this be not done it gets into the dust of rooms or cars, and lodges in carpets, blankets, curtains, and all upholstered furnishings where it can not be reached by any cleaning up process, however thorough, nor by any disinfectants other than in the form of gas; this alone can reach the deadly germs (tubercle bacilli), and destroy them in situ. In fact, in the interest of health, all carpets, rugs, and upholstered furnishings should be abolished. The bacilli of consumption, the cause of the disease, are very tenacious of life, and can only be destroyed by chemical means or fire. Sunlight, even the direct rays, will not kill them, nor will "fresh air," nor hot air, nor deodorizers, such as sheets wet with so-called disinfectants.

The question of limiting or arresting the spread of communicable disease is not solely a medical one; it is a medico-legal one. The medical men can only expound the causes and methods of propagation, and advise. They can preach the gospel of cleanliness, and expound the laws of hygiene, and recommend measures of prevention and protection, but they can not enforce them. The strong all causes in the registration area was 512,669. The latter only represents a little over one-third (28,000,000) of the total population. It is entirely safe to say that there were aproximately 150,000 deaths from tuberculosis in the United States every year from 1890 to 1900."

arm of the law must be invoked. Texas appreciates this fact, and it is a matter of pride and congratulation that she is first, and, so far, the only State in the Union to pass a law requiring disinfection (not simply "cleaning up") of public buildings and public conveyances in the interest of public health. This law went into effect July 1st, but is not yet in practical operation. Many of the railway surgeons of the State were here last week in conference with the State Health Officer as to the best methods and details, and it is understood that the State Health Officer is now preparing rules and regulations to be enforced in all public places for the protection of the public.

Meantime a concerted movement throughout the United States and Canada has been inaugurated by medical and legal scholars under the auspices of the Medico-Legal Society of New York, to stimulate a public sentiment and arouse the people, and, through them, the Legislatures and Congress to the necessity of protecting the public against this great and fatal danger not appreciated, indeed, scarcely suspected by the average citizen. The American Congress on Tuberculosis has been organized and will meet next spring in St. Louis during the great centennial. It will be held under the patronage of many of the most distinguished men, both medical and legal, in the United States, among whom are Secretary of State John Hay, Surgeon General Rixey of the navy, General Russell A. Alger, Surgeon General Nicholas Senn, the Earl of Minto (Governor General of Canada), all of whom, and many governors of States and statesmen are amongst the honorary presidents of the Congress. Its object is to put into operation every measure of prevention known to science, to secure the necessary legislation to this end, and to educate the people how they can avoid the disease; at least how the spread of the disease from the sick to the well may be measurably prevented by the observance and enforcement of sanitary precautions, both in families and in public. Thousands of lives will be saved annually by railway disinfection only.

The success with which sanitary science has dealt with yellow fever, eradicating it from its stronghold in Cuba, where, for two centuries, it has been endemic; with bubonic plague in Manila, and with cholera, that once dread scourge, warrants the hope and belief that in time it can greatly diminish the ravages of the great white plague, and by attacking the cause, as was done in the cases cited, it can finally be rendered practically harmless. It will require, however, a revolution in many details of public life and travel and in domestic life. Meantime much, a great deal, can be done towards limiting the spread of the disease and thus diminishing the fearful death loss. The people must be educated in sanitation. Its principles must be taught in our schools, and enforced at the same time. The law applies, of course, to public schools, and will, of course, be enforced.

The movement to create the American Congress on Tuberculosis was inaugurated by Hon. Clark Bell, long time President of the Medico-Legal Society and the well-known editor of the *Medico-Legal Journal*; and the successful organization of the Congress—despite opposition and even treachery on the part of certain medical men whose co-operation he had invited—is largely, mainly, due to his intelligent zeal in the cause. The medical profession owe him much; the American people more. His work in this alone will be a monument to his name, and history will give him a place by the side of Pinel, Parr, Howard and other great humanitarians and philanthropists.

